# Influence of Gutta-Percha Surface on *Enterococcus faecalis* Initial Adhesion In Vitro: An Atomic Force Microscopy Study

**DOI:** 10.3390/life13020456

**Published:** 2023-02-06

**Authors:** Allan Victor Ribeiro, Evelyn Giuliana Velásquez-Espedilla, Mirela Cesar de Barros, Letícia Lobo de Melo Simas, Flaviana Bombarda de Andrade

**Affiliations:** 1 Department of Physics, Federal Institute of São Paulo, Birigui CEP 16201-407, SP, Brazil; 2 Department of Dentistry, Endodontic and Dental Materials, Bauru School of Dentistry, University of São Paulo, Bauru CEP 17012-901, SP, Brazil

**Keywords:** *Enterococcus faecalis*, gutta-percha, microscopy atomic force, bacterial adhesion, topography surface

## Abstract

The aim of this study was to evaluate the influence of surface topography of gutta-percha (GP) cones and plasticized disks of GP on the initial adhesion of *Enterococcus faecalis* (*E. faecalis*). The GP cones (Tanari and Dentsply brands) were cut 3 mm from the apical portion and fixed on a glass slide. To make the disks, the cones were thermoplasticized in standardized molds. The specimens were divided into groups according to the shape of the GP and the presence or absence of the bacteria. For contamination, the strain of *E. faecalis* (ATCC 29212) was used. The surface topography was analyzed using an atomic force microscope (AFM). The surface, roughness, and waviness parameters were evaluated by the Kruskal–Wallis and Dunn test. The comparison between disks and cones showed significant differences, where the cones were rougher, with a higher value attributed to the Dentsply cone (DC group). The same was observed for the waviness. After contamination, there was greater bacterial accumulation in cones, especially in their valleys, but both the surface and the topography became more homogeneous and smoother, with no differences between disks and cones of both brands. The topographic surface of the GP, at the micro and nanoscale, influences the initial adhesion of *E. faecalis*, with a greater tendency for contamination in regions associated with the presence of roughness and waviness. In this context, plasticization of GP is indicated, as it reduces surface irregularities compared to cones, contributing to less retention of bacteria.

## 1. Introduction

The success of endodontic treatment depends not only on maximally eliminating the microbial load inside the root canal system (RCS), but also on sealing the apical and coronal pathways to avoid potential fluid infiltration and maintain the state of disinfection achieved by chemical and mechanical cleaning, preventing reinfection, percolation of bacterial substrates, and allowing the periodontium to achieve healing [1,2].

Gutta-percha (GP), one of the most used and accepted materials for filling root canals, is a natural polymer extracted in the form of latex and used in the manufacture of cones and sticks. The chemical composition and the number of inorganic substances added, as well as the thermal changes induced during the cones’ manufacturing process, affect the properties of the material, conferring advantages that favor its use in the obturation of root canals [1]. The primary components of gutta-percha cones are zinc oxide (75%) and gutta-percha (20%). The remaining components are barium sulfate, resins, and coloring agents [2].

Resilon (Resilon; Resilon Research LLC, Madison, CT, USA), a polymer-based synthetic thermoplastic material, was launched to challenge gutta-percha as a solid root canal filling material. Resilon’s thermoplasticity is provided by polycaprolactone, a biodegradable polyester with a moderately low melting point, while its bonding is derived from the resin’s inclusion with methacryloxy groups. This filler, which contains glass fillers and barium chloride as fillers, is also capable of coupling to resin sealers. In addition, it is similar to GP in size and some diameters, although less used [3].

Even though GP cones are manufactured under aseptic conditions, they can be contaminated during handling, by aerosols, and from physical sources during storage [3]. Due to their plastic characteristics, they cannot be sterilized by conventional techniques (autoclave or dry heat), which would cause changes in their structure and consequent dimensional changes, representing a great potential for failure in endodontic obturation [3,4]. Additionally, after root canal filling, if there is a failure in the coronal sealing and consequent infiltration of fluids, this gutta-percha can serve as a substrate for microbial proliferation.

One of the possible ways to prevent these failures is the decontamination of GP cones before obturation to resist the formation of biofilm on its surface. For this, different antimicrobial solutions can be used, such as sodium hypochlorite (NaOCl), alcohol, iodine compounds, hydrogen peroxide, and natural compounds [3,5,6,7,8]. Although NaOCl is the disinfectant of choice most of the time, studies indicate that higher concentrations, such as 5.25%, or the exposure time cause topographic changes in the cones [9]. In this context, alternatives such as chitosan, propolis, ultraviolet C (UVC), and ozone have been studied. They have shown promising results in terms of antimicrobial action and maintaining the topographic and physical properties of the materials with which they come into contact [10,11].

Atomic force microscopy (AFM), a member of the scanning probe microscope family, is a well-established and documented tool for structural characterization of materials [6]. The growing interest in using it in investigations of this type is due to its ease of use, relatively low cost, molecular level resolution, and the variety of surfaces it can examine. Applications include 3-dimensional images of various materials, such as metals, polymers, ceramics, crystals, minerals, biomolecules, and cells, with high spatial resolution and evaluation of mechanical properties. Its working principle is through probing the surface of the sample with a small tip attached to a flexible cantilever, generating maps of topography and physical properties. The three-dimensional nature of the obtained images allows the measurement of structural features and the generation of structural statistics [7,8].

Bacterial adhesion to a surface with consequent biofilm formation is a complex process that occurs in stages being influenced by the types of interaction and physicochemical characteristics of the substrate. In the initial stages of biofilm formation, this dynamic occurs in terms of colloidal interactions, where the adhesive property of bacterial cells plays a major role for irreversible attachment, as well as chemical composition, wettability/hydrophobicity, and aspects related to topography and surface roughness [12,13,14,15]. Each of these variables can have different degrees of relevance in the biofilm formation process [16], impacting the adhesion strength of bacteria to the surfaces of biomaterials [17].

The relationship between roughness and bacterial adhesion has been extensively studied [12,14,15,18,19]; however, recent literature is limited to amplitude parameters such as mean roughness (RMS) and surface mean roughness (Ra) [3,7,8], not characterizing the shape of the peaks or how the surface irregularities are spatially distributed. In this sense, it is necessary to analyze a larger set of topographic parameters that can provide additional elements on the shape and spatial distribution of surface irregularities, which directly affect the initial deposition of bacteria under static conditions [8,13,20].

*Enterococcus faecalis* (*E. faecalis*), one of the most commonly isolated bacterial species from unsuccessful endodontic root canals, is a facultative Gram-positive bacterium [21]. It has the ability to invade dentinal tubules, form biofilms, and survive the adverse environmental conditions present in the root canals of endodontic treated teeth, it can survive endodontic irrigation and high concentrations of intracanal drugs, as well as large pH variations [22,23]. Microorganisms that survive the chemical–mechanical preparation of the RCS or persist in the remaining filling materials (gutta-percha/sealer) may influence the prognosis of endodontic treatment in favorable circumstances [23].

Although the average roughness (Sa) and RMS (Sq) parameters are commonly used to characterize the surface of materials, in studies involving bacterial adhesion, they are insufficient to evaluate relevant aspects of the topography that impact adhesion. Thus, the present study aimed to evaluate the influence of the topographic surface of GP cones and plasticized disks on the initial adhesion of *E. faecalis*, using conventional and additional parameters of surface roughness.

## 2. Materials and Methods

### 2.1. Specimens Preparation

GP cones ISO size 40, taper 0.04 (Dentsply Petrópolis, RJ, Brazil), and ISO size 70 (Tanari, Tanariman, Manacapuru, AM, Brazil) were used to prepare the specimens. Each GP cone was cut 3 mm from the apical portion and fixed to a glass slide using quick-setting cyanoacrylate glue [6]. To make the GP disks (6.3 mm in diameter × 1.7 mm in thickness), the GP cones were heated in a water bath on a sterilized glass surface at 70 °C, monitored by a digital thermometer. Then, the heated materials were placed in standard metal ring molds, with the dimensions described above, and pressed between two glass plates under constant controlled pressure of 0.5 N for 1 min [24]. Then, the samples (disks and cones) were decontaminated in UV light inside a laminar flow chamber (Esco, Airstream, class II A2, Jacareí, São Paulo, Brazil) for 30 min on each side.

### 2.2. Experimental Groups

The eight evaluated groups were divided as follows, each one containing 12 samples:

TD–GP disk (Tanari, Brazil);

DD–GP disk (Dentsply Sirona, Brazil);

TC–GP cones (ISO size 70, Tanari, Brazil);

DC–GP cones (ISO size 40, taper 0.04, Dentsply Sirona, Brazil);

TD_B_–GP contaminated disk (Tanari, Brazil);

DD_B_–GP contaminated disk (Dentsply Sirona, Brazil);

TC_B_–GP contaminated cones (ISO size 70, Tanari, Brazil);

DC_B_–GP contaminated cones (ISO size 40, taper 0.04, Dentsply Sirona, Brazil).

### 2.3. Bacterial Contamination Protocol with Enterococcus faecalis

**Figure 1:**
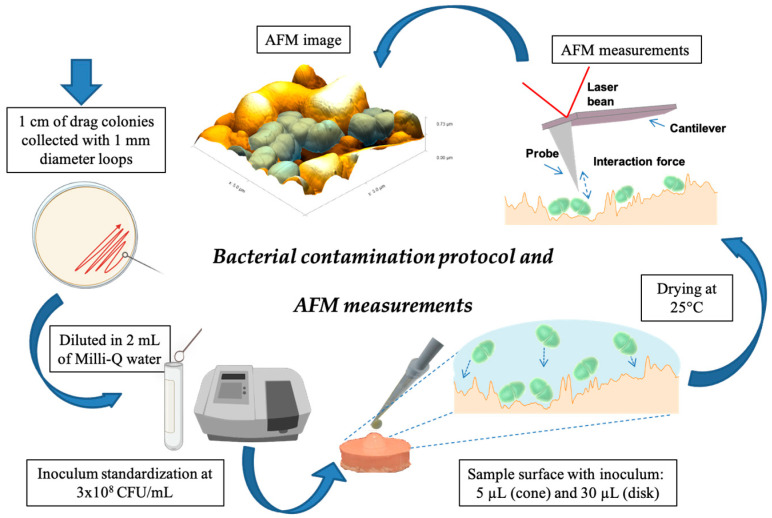
Schematic representation of the contamination protocol and AFM measurements.

A strain of *E. faecalis* (ATCC 29212) (American Type Culture Collection) was reactivated in BHI broth (Difco, Kansas City, MO, USA) and kept at 37 °C for 24 h. Purity was confirmed by Gram stain (Oxoid, Basingstoke, UK) and bacterial morphology. The culture was adjusted to a concentration of 3 × 10^8^ CFU/mL (standard n° 1 of the McFarland scale) using a spectrophotometer (Shimadzu UV-1800, Kyoto, Japan) and then plated. For the contamination of the GP samples (cones and disks), 1 cm of drag colonies grown on BHI agar collected with 1 mm diameter loops, diluted in 2 mL of Milli-Q water, were used. Pipetting of this inoculum was 5 µL for the cones and 30 µL for the GP disks. The experimental procedures were performed by a single operator inside a laminar flow chamber. After drying time at 25 °C, 15 min for the Tanari group and 30 min for the Dentsply group, the samples were analyzed in AFM. The different brands presented different drying times for inoculum. This time was standardized in a pilot study to observe only countable bacterial cells on the samples, seen in the images. The schematic representation of the contamination protocol and AFM measurements is indicated in Figure 1.

### 2.4. AFM Analysis

The samples were submitted to topographic analysis by AFM (FlexAFM-NanoSurf AG, Liestal, Switzerland, with C3000 controller with ADC and 24-bit DAC) being performed for three measurements per sample: three different regions of the disks and between 1 mm and 3 mm from the tip of the cones [6]. Measurements were performed in tapping mode (Dynamic Force) at a rate of 0.5–1  Hz. The tips used were Tap190Al-G-10 (BudgetSensors, Sofia, Bulgaria) with a radius of less than 10 nm. The cantilever used has a force constant of 48 N/m (28–75 N/m), a resonance frequency of 190 kHz (nominal values provided by the manufacturer), and was positioned parallel to the cones. Scanned areas were uniformed squares of 5 µm × 5 µm. The images and topographical parameters were obtained using the FlexAFM-NanoSurf atomic force microscopy system, with a resolution of 512 × 512 pixels, and analyzed using the Gwyddion software (version 2.55, Czech Metrology Institute, Brno, Czech Republic). The background of the slopes was fixed.

For comparison purposes, mean roughness (Sa), root mean square (Sq), and maximum surface height (Sz) were chosen to investigate the topographic surface of GP cones and disks. The profile roughness at 2.5 µm, Ra and Rq, and the waviness parameters Wa (waviness average) and Wq (waviness RMS) were also measured. In addition, the projected area was measured to determine the number of adhered *E. faecalis*.

### 2.5. Statistical Analysis

Data distribution was assessed using the Shapiro–Wilk normality test. Therefore, for intergroup comparisons regarding the observed parameters, the Kruskal–Wallis followed by the Dunn test was performed. To evaluate the adhesion of *E. faecalis* among the contaminated groups, the one-way ANOVA test followed by the Dunn test was performed. Prism 6.01 software (GraphPad Software, Inc., San Diego, CA, USA) was used for the analyses, adopting a significance level of 5%.

## 3. Results

**Figure 2:**
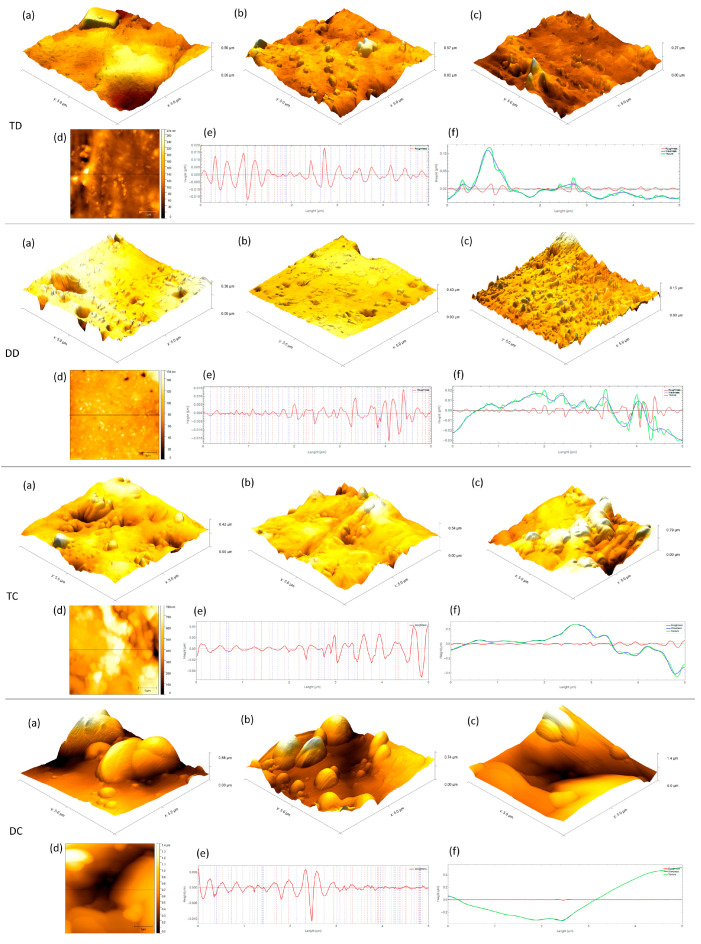
AFM images of the topographic surface of the different groups without contamination. (**a**–**c**) 3D (different samples) and (**d**) 2D topography. (**e**) In the roughness profile at 2.5 µm, the positions of peaks (red) are indicated. (**f**) The texture (green), waviness (blue), and roughness (red) graphics are presented.

Three-dimensional AFM images of GP cones and disks are shown in Figure 2. Analysis of the surface topography showed qualitative and quantitative differences between the uncontaminated groups. For Sa and Sq, the comparison between disk and cones showed a significant difference (*p* < 0.05), with the cones being rougher in all crossings and the highest value attributed to the DC group. For Sz, only the DC and TC groups showed significant differences, with the highest values attributed to DC (*p* < 0.05).

**Figure 3:**
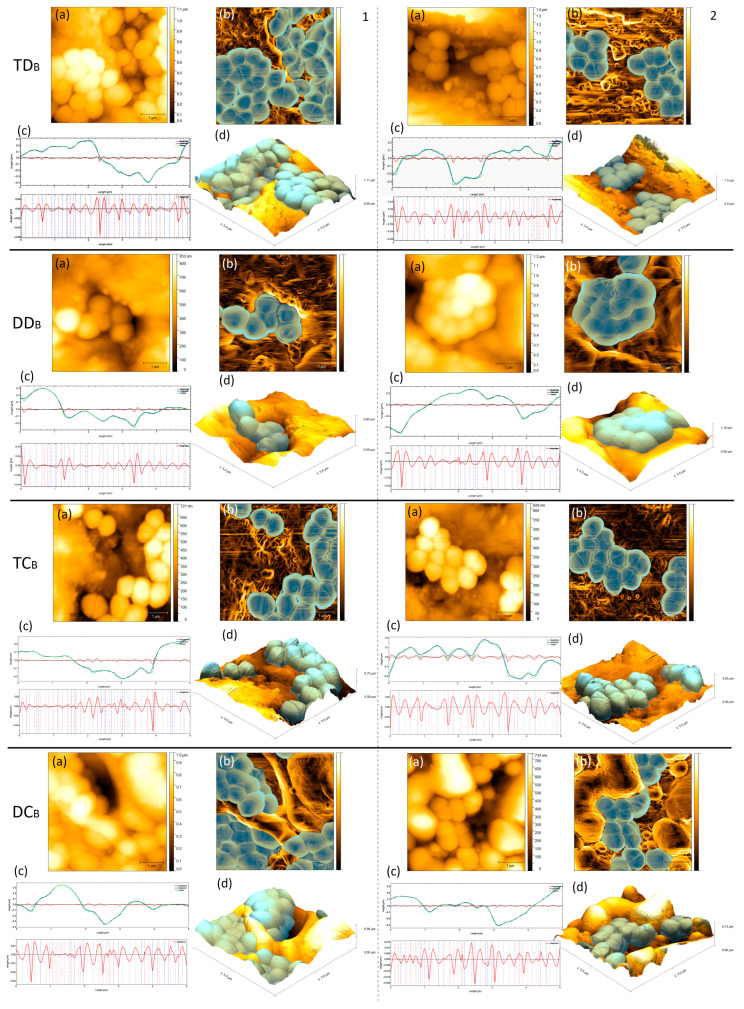
AFM images of the topographic surface of the different contaminated groups. Two different AFM images (1 and 2 column) are presented for each group. (**a**) 2D topography (**b**) 2D in presentation mode with edge detection by inclination and (**d**) 3D AFM image are presented. *Enterococcus faecalis* is indicated in blue. (**c**) The texture (green), waviness (blue), and roughness (red) graphics (top) are presented. In the roughness profile at 2.5 µm (bottom), the positions of peaks (red) and valleys (blue) are indicated.

Among the contaminated groups, only DD_B_ and TC_B_ showed significant differences (*p* < 0.05) with higher roughness values, Sa and Sq, for DD_B_ (Table 1). Figure 3 shows the average amount of *E. faecalis* adhered to the contaminated samples and the average area covered. Among the disks and cones evaluated, only the TD_B_ and TC_B_ groups showed significant differences, with the highest and lowest amount of adhered bacteria, respectively (*p* < 0.05).

Table 2 presents the median, minimum, and maximum of the roughness profiles of the analyzed groups. TC and DC had the highest values, as well as the highest number of adhered bacteria. Regarding the waviness, the comparison between the different groups showed that the cones (TC and DC) presented higher values than the disk groups (TD and DD) (*p* < 0.05). All contaminated groups were similar with no statistical differences (*p* > 0.05).

## 4. Discussion

The main objective of endodontic treatment is to prevent or treat apical periodontitis and to reach this, cleaning, modeling, disinfection and three-dimensional filling of the RCS are necessary [25]. It is important that the filling material has the ability to stimulate the repair of periapical tissues, be bactericidal, dimensionally stable, biocompatible, and promote sealing of the canal system, preventing microleakage and maintaining the asepsis reached by chemical–mechanical preparation [26].

Even when these procedures are successfully performed, bacteria can survive the inhospitable environment created by the biomechanical preparation, forming biofilms and still adhering to biomaterials, representing a challenge to the success of endodontic treatment [17]. Recent studies point to the high adhesion capacity of *E. faecalis* in GP [27,28] and in sealers used to fill root canals [28,29,30]. In endodontics, the biofilm adhered on the biomaterial can be intraradicular or extraradicular, depending on the position of the filling material. Takemura et al. [31] suggested that Gram-positive facultative anaerobes can adhere and form biofilms on GP cones, regardless of the type and technique of obturation.

Dentists are occasionally facing the problem of infections that occur after filling the root canal space. This fact may be related to the introduction of contaminated GP cones in the root canal. In this sense, to avoid contamination of this material, they must be treated with decontaminating agents before filling the canals. NaOCl is the most used solution due to its broad spectrum of action against a variety of Gram-positive, Gram-negative, and spore-forming bacteria [32,33]. However, factors such as time, chemical nature, and concentration of the chosen solution must be taken into account in order to avoid deterioration of the surface topography, which corroborates the increase in roughness, favoring bacterial adhesion [34]. Kubasiewicz-Ross et al. [32] evaluated different forms of decontamination of the implants’ surface and observed that the treatments that promoted greater decontamination were responsible for increasing the roughness of the material. Based on that, the search for alternative methods of disinfection is highly recommended in order to reconcile decontamination with the preservation of the surface topography of the material.

The choice of *E. faecalis* is associated with its prevalence in cases of persistent or secondary infection. It is an opportunistic bacterium that has an ovoid shape, between 0.5 to 1 µm in diameter, and occurs individually, in pairs, or in short chains, changing its physicochemical properties according to the prevailing environmental and nutritional conditions. In addition, these bacteria have the ability to adhere to dentin collagen and form biofilms, which contribute to bacterial persistence even after endodontic procedures [35,36]. As a resistance mechanism, they can reach a viable but non-cultivable state, activating a starvation response under stress conditions, which allows reinfection of the filled root canal [37]. Besides the mentioned reasons, the authors sought to simulate a possible clinical condition of a correctly filled root canal, but which, for some reason, was contaminated, in order to assess which form (cone or plasticized) the gutta-percha would be less favorable to bacterial adhesion and subsequent recontamination.

AFM, a powerful surface characterization tool, provides three-dimensional images of molecular surfaces with nanometer resolution and does not require any special treatment that could lead to sample deterioration. This equipment has been widely used to evaluate the micro and nanoscale topography of different substrates, including gutta-percha cones [6,8]. In this work, we present AFM images that are rich in detail and of superior quality to those presented previously [3,7,38], in addition to investigating the influence of GP surface topography on the initial adhesion of *E. faecalis*.

Surface roughness and its topographic characteristics are critical factors that physically affect bacterial adhesion to surfaces and have been extensively studied [6,8,18,19,30]. It is known that the literature points to other surface characteristics, including surface free energy and substrate hydrophobicity, which also influence this phenomenon [28]; however, despite the large volume of data available on the subject, there is no consensus. Most bacterial adhesion studies normally consider roughness parameters that describe only one aspect of surface topography such as surface mean roughness (Ra) and root-mean-square roughness (RMS); however, they do not provide information on the spatial distribution [6,7]. Before contamination, cones appear rougher than disks, and it was possible to observe a significant difference in the surface topography of the analyzed groups, mainly for the cones, which are hardly characterized, only by Sa and Sq, justifying the need to adopt complementary parameters such as Sz and waviness (Wa and Wq). Differently from what was observed, Wu et al. [13] and Crawford et al. [19] indicated a lack of direct correlation between surface roughness and bacterial initial adhesion.

In comparison between disks and cones, the evaluated parameters indicated higher values for the cones, showing that the plasticization of the GP contributed to the smoothing of the surface and related to the lower roughness (Sa, Sq) and waviness (Wa, Wq) (Table 1 and Table 2). In general, great variability was observed in the surface topography. The same was verified in previous works [3,6,7,8], which can be justified by the analysis in different positions in the same sample or different samples. To overcome this variation, Prado et al. [39] suggested the standardization of the scanning position of the samples, however, to carry out the contamination protocol and to maintain this position becomes difficult.

The influence of surface topography on the adhesion propensity of *E. faecalis* cells has been previously investigated [40]. It is suggested that microbial adhesion to a substrate occurs in two distinct phases. Phase 1 is the initial interaction (in seconds to minutes), which occurs when the cell approaches the surface of the substrate, according to the distance between them. Phase 2 (in hours to days) occurs between surface structures of the microbial cell, in which adhesion molecules and/or virulence factors are expressed [13,17]. The first step for the formation of a biofilm on a substrate is the presence of a microorganism associated and with interaction with the surface and its physicochemical characteristics [12]. A correlation between the different stages of biofilm development with changes in the average surface roughness was proposed, in agreement with our results, which showed an increase in the roughness parameters in all contaminated samples. Among the evaluated brands, the Tanari cones (TC) and the Dentsply cones (DC) showed higher roughness and waviness and a large number of adhered bacteria, however, without significant differences between them (*p* > 0.05). Thus, it is suggested that in addition to the surface topography, the roughness profile and the waviness influence bacterial adhesion numbers between the samples studied.

Bacteria appear to try to maximize surface contact area to obtain a stronger and more stable attachment, which results in a specific alignment depending on the arrangement and topographic details. It was also verified that in their initial adhesion, bacteria are mostly grouped in small clusters close to valleys and within them. Hsu et al. [41] found a similar behavior for bacterial adhesion on controlled topographical surfaces, where, in regions of lower roughness, adhesion was small, compared to regions with accentuated irregularities. In agreement, Linklater et al. [30] demonstrated that bacteria preferentially adhere to valleys and spaces on microtextured substrates, colonizing only recessed regions that are larger or approximately equal to their own size. The AFM images (Figure 3) indicated several topographic areas of the sample surfaces that may be within the same size scale as *E. faecalis*, increasing bacterial adhesion (Table 1). In fact, the presence of irregularities on the surfaces of the GP specimens, in particular the cones, may represent a great potential for endodontic filling failure, as previously reported [6], influencing the quality of the filling by providing space for the percolation of fluids that will serve as nutrients for the surviving microorganisms in the RCS.

It is important to emphasize that the previous roughness of the samples will influence bacterial adhesion/retention, evidenced in the DC and TC groups. With the use of AFM, it was possible to observe and evidence the need for standardization of the GP cones on a microscopic scale, providing a smooth and uniform topography, what we can obtain by thermoplasticized techniques. The results of this study provide evidence that the topographic surface of the GP, at the micro and nanoscale, influences the initial adhesion of *E. faecalis*, with a greater tendency of this occurrence in irregular regions associated with the presence of roughness and waviness. It also provides evidence of the influence of the surface nano-architecture of the substrates on the attachment of bacteria, but more investigations are recommended evaluating physical–chemical and wettability properties of the biomaterials concerning bacterial adhesion. In addition, we also highlight the originality of the present work when evaluating gutta-percha, a filling material for the root canal, in the conventionally used form (cones) and plasticized (disk), not only to understand the influence of the physical state of this material in the recontamination of the root canal but also suggesting to professionals the adoption of techniques that further increase the success rates of endodontic treatment.

As limitations of this study, we highlight the number of AFM readings/measurements performed per sample (only three) and profile lines per image. The time necessary to perform the readings in high resolution, takes around 20–30 min, which is considered long, taking into account that *E. faecalis* has a relatively short proliferation time. At the same time, a larger number of readings would result in more information about the topographic surface but also would result in a significant increase in the time and in the counting of proliferating bacteria adhered to the analyzed substrate, which could provide mistaken data. Complementary measurements related to the physical–chemical characterization of the surface and quantification of the adhesion strength of *E. faecalis* are important to understanding the adhesion process and need to be carried out in future studies. As the focus and objective of this study, we present evidence on the necessity to adopt and standardize other topographic parameters, little discussed in the literature, and how they can contribute to a better understanding of how the nature of the substrate influences bacterial adhesion to the substrate.

## 5. Conclusions

The micro and nanoscale surface topography of GP affects the initial adhesion of *E. faecalis*, favoring its occurrence mainly in valley regions, frequent in rough and wavy surfaces. The topographic characteristics had a decisive influence on the adhesion process; therefore, in general, the disks, or plasticized gutta-percha, were less irregular, accumulating less bacteria, a fact that must be taken into account in clinical practice, in order to make the recontamination of the obturated root canal difficult.

## Figures and Tables

**Table 1:** Quantitative analysis of the topographic surface parameters in median, minimum, and maximum.

Groups	RMS Roughness (Sq) nm	Mean Roughness (Sa) nm	Maximum Height (Sz) nm
TD	56.86 (22.37–63.91) ^Aa^	37.03 (16.43–48.28) ^Aa^	453.4 (275.9–569.8) ^Ab^
DD	33.53 (22.92–37.59) ^Aa^	25.8 (16.94–27.24) ^Aa^	308.6 (211.6–390.7) ^Ab^
TC	71.51 (58.21–82.01) ^ACa^	51.41 (42.73–61.03) ^BCa^	561.5 (478–707.3) ^BCb^
DC	173 (132.3–253.7) ^Ba^	142.6 (102.1–186.7) ^Ba^	883.7 (686–1389) ^BCb^
TD_B_	132.7 (113.4–212.5) ^ACa^	108.7 (90.43–163.5) ^ACa^	762.1 (611.6–1309) ^ABb^
DD_B_	199.5 (162.9–217.7) ^Ba^	154.4 (133.6–185.4) ^Ba^	1168 (868.7–1327) ^BCb^
TC_B_	134.1 (120.1–144.6) ^BCa^	107.4 (97.73–117.8) ^BCa^	724 (623.4–810.1) ^Bb^
DC_B_	149 (120–189.1) ^BCa^	119 (98.58–146.7) ^BCa^	937.1 (723.1–1093) ^Bb^

Kruskal–Wallis and Dunn test (*p* < 0.05). Different superscript capital letters in a column represent significant differences between groups; different superscript lowercase letters on a line represent significant differences within groups.

**Table 2:** Quantitative analysis of the profile roughness parameters and waviness in median, minimum, and maximum.

Groups	RMS Roughness (Rq) nm	Mean Roughness (Ra) mm	Waviness Average (Wa) nm	RMS Waviness (Wq) nm
TD	4.30 (2.47–6.39) ^Aa^	3.21 (1.94–4.87) ^Aa^	21.8 (12.58–39.86) ^Ab^	30.25 (15.83–51.19) ^Ab^
DD	3.47 (2.8–4.55) ^Aa^	2.32 (2.04–2.91) ^Aa^	14.69 (13.52–23.96) ^Ab^	18.73 (16.22–32.82) ^Ab^
TC	6.27 (4.22–8.86) ^BCa^	4.33 (3.06–6.32) ^Ba^	58.51 (46.36–94.67) ^BCb^	77 (58.22–112.4) ^ABb^
DC	4.7 (1.62–6.7) ^ABa^	3.39 (1.09–4.63) ^ABa^	118.3 (85.41–205.6) ^Cb^	138.5 (99.94–237.8) ^BCb^
TD_B_	11.89 (7.62–14.75) ^ABa^	8.64 (5.28–11.01) ^ABa^	119.3 (78.65–156.7) ^ABb^	138.3 (94.35–192.7) ^BCb^
DD_B_	7.1 (5.2–8.1) ^Aa^	4.87 (3.52–5.97) ^Aa^	164 (94.53–190.7) ^BCb^	181.3 (115.1–215.3) ^BCb^
TC_B_	9 (6.92–13.54) ^BCa^	6.56 (5.22–10.48) ^Ba^	112.8 (95.12–133.8) ^BCb^	130.9 (116.4–148.9) ^BCb^
DC_B_	7.33 (6.3–9.08) ^ABa^	5.54 (4.73–6.81) ^ABa^	102.7 (84.68–125.7) ^BCb^	119.5 (109.8–163.1) ^BCb^

Kruskal–Wallis and Dunn test (*p* < 0.05). Different superscript capital letters in a column represent significant differences between groups; different superscript lowercase letters on a line represent significant differences within groups.

## Data Availability

Data sharing is not applicable for this article.
